# Complex Mandibular Reconstruction for Head and Neck Squamous Cell Carcinoma—The Ongoing Challenge in Reconstruction and Rehabilitation

**DOI:** 10.3390/cancers12113198

**Published:** 2020-10-30

**Authors:** Tomislav A. Zrnc, Josip Tomic, Peter V. Tomazic, Hamid Hassanzadeh, Matthias Feichtinger, Wolfgang Zemann, Philipp Metzler, Mauro Pau

**Affiliations:** 1 Department of Oral and Maxillofacial Surgery, University Hospital, Medical University of Graz Auenbruggerplatz 5, 8036 Graz, Styria, Austria; josip.tomic@medunigraz.at (J.T.); matthias.feichtinger@medunigraz.at (M.F.); wolfgang.zemann@medunigraz.at (W.Z.); philipp.metzler@uzh.ch (P.M.); mauro.pau@klinikum-graz.at (M.P.); 2 Department of General ORL, Head and Neck Surgery, University Hospital, Medical University of Graz, Auenbruggerplatz 26, 8036 Graz, Styria, Austria; peter.tomazic@medunigraz.at; 3 Department of Orthopaedic Surgery, University of Virginia Health System, Charlottesville, VA 22903, USA; HH4XD@hscmail.mcc.virginia.edu

**Keywords:** HNSCC, head and neck squamous cell carcinoma, surgical treatment, mandible composite reconstruction, free flap, prosthetic rehabilitation, quality of life, EORTC QLQ-C30, EORTC QLQ-H&N43

## Abstract

**Simple Summary:**

Cancer therapy includes a broad range of microvascular free flaps that may restore defects and improve patients’ quality of life. This is particularly important for head and neck squamous cell carcinoma (HNSCC) and composite mandibular reconstructions, containing tissues of bone, muscle, and skin, which may be problematic due to their magnitude and sensitive location. The subscapular system offers a highly valuable donor site with the most versatility and the potential for rapid rehabilitation. Interestingly, other donor sites are more commonly used internationally. Therefore, we evaluated the use of the subscapular system free flap (SFF), which is the most commonly used free flap at our department. To our knowledge, this retrospective study represents the largest number of SFF cases reported to date in the literature. Furthermore, we examined the quality of life in a subgroup of patients, combining prospective occurrences to provide insight into overall rehabilitation from the patients’ viewpoints.

**Abstract:**

Large head and neck squamous cell carcinoma (HNSCC) tumors affecting the mandible require a versatile reconstruction to maintain form, function, and quality of life. Large defect reconstruction of soft and hard tissue in the head and neck necessitates, at best, one vascular system including various tissues by large dimensions. The subscapular flap system seems to meet these standards. A retrospective study was conducted focusing on clinical data, including an analysis of the quality of life with the European Organization for Research and Treatment of Cancer (EORTC) Quality of Life Questionnaires, (QLQ-C30 and QLQ-H&N43). A total of 154 patients (122 males, 32 females; age range: 31–71 years, mean: 54.5 years) treated at our department from 1983 through to 2019 were included. Of the subscapular system free flaps (SFFs), 147 were based on the angular artery branch of the thoracodorsal pedicle (95.45%), and the remaining seven cases (4.55%) were lateral scapular border flaps. Mean mandible defect length was 7.3 cm. The mean skin paddle dimension was 86.8 cm^2^. The most common recipient artery was the thyroid superior artery (79.22%). Major postoperative complications occurred in 13 patients (8.44%). This study confirms that SFFs offer excellent soft and hard tissue quality, component independence, a large arc of rotation length, and a large gauge of pedicle, making them the gold standard for the reconstruction of large composite defects of mandibular HNSCC tumors.

## 1. Introduction

In 1973, the first valid microsurgery technique was performed that offered the opportunity to perform free flaps [1,2]. This surgical development was a real turning point in reconstructive surgery. Baudet et al. (1976) reported the first free flaps harvested from the axillary region [3]. Meanwhile, there was a growing interest in microvascular grafts from various anatomical regions, especially the subscapular system [4,5]. The first studies of bone flaps explained that the bone feeder originated at the lateral border of the scapula, supplied by a branch of the circumflex scapular artery [6,7]. Knowledge of the local vascular system offers insurance against flap failure. In the late 1980s, the angular branch in particular was described by Deraemaecker et al. as an additional blood supply to the lower lateral border of the scapular bone [8]. This finding led to a better understanding of the mobility of soft and hard tissues, resulting in improved length and a suitable arc of pedicle rotation of osteocutaneous flaps based on the thoracodorsal vessels [8,9,10,11]. In 1991, Coleman and Kärcher described the first subscapular system free flap (SFF) based on the angular branch of the thoracodorsal artery for mandibular reconstruction [9,10].

Although the versatility of the subscapular system for mandibular reconstruction was well noted, it was the development of deep circumflex iliac artery (DCIA) flaps and fibula free flaps (FFFs) during the 1990s that caused a decline in the popularity of SFFs [5]. Among the bone flaps, these flaps have favorable morphologic configurations, allowing for good anatomic bone reconstruction of the mandible [5]. At present, SFF is the second or even third choice for the reconstruction of soft and hard tissues [5]. However, valid reconstruction of large-volume oromandibular defects with a DCIA flap or an FFF can only be achieved using an additional free flap. Inevitably, this leads to a prolonged operative time, hospital stay and recovery period, and additional risk of morbidity and complications. In particular, SFFs are extremely advantageous in this case [5,12,13]. Here, the variability of the vascular subscapular system plays a role as well, especially due to its wide mobility, the large variety of different tissues, and the unique potential of flap tissue with a large surface area. Furthermore, the branching pattern of the subscapular system enables the transferal of select parts of the scapula, the latissimus dorsi, the myocutaneous para- and scapular flaps, and even parts of the serratus and ribs pedicled on one single vessel. In fact, these characteristics make it the most versatile of all microvascular composite flaps, especially for complex cranio-maxillofacial defects of various quality (bony, fat, muscle, cartilage, and skin) and quantity [5,12,13].

Since 1983, the DCIA flap and FFF have been the workhorse flaps for bone reconstruction of mandibular defects at our department. However, subscapular SFFs have been the main choice for the reconstruction of large and complex composite defects, especially for large head and neck tumors. In addition, a quality of life (QoL) survey of this patient cohort was implemented. However, an explanation of the concept of QoL with all its facets is necessary to create a uniform basis of definition, and to make it possible to achieve comparable results in similar scientific work. The WHO defines health as “a state of complete physical, mental and social well-being”. Being free from disease does not therefore automatically mean being healthy. QoL is a multidimensional product of physical, mental, and social components, and thus shows parallels to the WHO’s definition of health [14]. The concept of health-related quality of life (HRQoL) aims to evaluate the above-mentioned components as parameters after a medical treatment (e.g., an operation). For many patients, the focus is not only on extending their lifetime, but also on their ability to continue living their lives in a fulfilled manner. The focus is often on the fear of not being able to return to a familiar life due to restrictions in aesthetics and physical function, and the associated social exclusion. The head and neck area in particular is considered to be a sensitive area where surgical interventions can cause severe limitations [15]. The primary goal should therefore be to enable patients to enjoy an unlimited sense of well-being in all areas of life after a therapeutic intervention. In the sense of the biopsychosocial model, which represents a connection between illness, body, mind, and social interaction [16], HRQoL includes aspects of the concept of health as defined by the WHO. A definition of HRQoL consistent with the WHO’s definition of health is “how well a person functions in life based on perceived well-being in physical, mental and social areas of health [17]”.

During the last few decades, HRQoL has become increasingly important as an assessment parameter in evidence-based medicine. This is evident in literature databases such as PubMed, where a strong increase in publications dealing with HRQoL has been recorded (PubMed Timeline 1961–2020).

To be able to assess the patient’s condition for follow-up and therapy evaluation, appropriate measuring instruments in the form of questionnaires are required [18]. Among the best-known measuring instruments used are the Short-Form 36 (SF-36) and the University of Washington Quality of Life Questionnaire (UW-QOL) [19], as well as the questionnaires developed by the European Organization for Research and Treatment of Cancer (EORTC), which are currently considered the gold standard and were used in this study [15].

The aim of this study was to share our experience of the reconstruction of large composite mandibular defects involving soft and hard tissues; thereby, we sought to demonstrate the versatility and reliability of this type of free flap reconstruction in a number of patients. In addition, for the first time, we present a QoL survey of a subgroup reconstructed with the SFF. To the best of our knowledge, this study represents the largest survey of head and neck squamous cell cancer patients who underwent mandibular reconstruction with SFFs.

## 2. Results

Complete data were evaluated from 154 patients within the 35-year period from December 1983 to December 2019. The study included 122 male (79.22%) and 32 female patients (20.78%), with a mean age of 54.4 years and ranging from 31 to 71 years old. All patients underwent segmental mandibular resection and immediate reconstruction with an SFF. Histopathological diagnosis was positive for head and neck squamous cell carcinoma (HNSCC). Mandibular defects were classified according to the scheme proposed by Urken et al. [20]. The bone defect involved only one segment of the mandible in 27 patients (17.53%). Tumor resection and the reconstruction of two segments was performed in 93 patients (60.40%). Twenty-five patients (16.23%) underwent resection of three mandibular segments. In seven patients (4.54%), the defect consisted of four mandibular segments. One patient (0.65%) underwent reconstruction of a five-segment defect and one patient (0.65%) a six-segment defect.

Concomitant neck dissection was performed in 133 patients (86.36%). Of these, 128 underwent ipsilateral (83.11%) and the remaining five (3.25%) underwent both ipsilateral and contralateral neck dissection. Furthermore, 65 were radical neck dissections (RNDs) (42.20%), eight (5.20%) were type III modified radical neck dissections (MRNDs), and 60 (38.96%) were selective neck dissections (SNDs).

The pedicle of the scapular was based on the angular artery and vein in 147 grafts (95.45%), and the remaining seven cases (4.55%) were lateral scapular border flaps. The length of the bone graft ranged from 4.2 to 15.6 cm (mean 7.3 cm). The dimensions of the latissimus dorsi skin paddles ranged from 14.3 to 250.8 cm^2^ (mean size of 86.8 cm^2^).

The majority of recipient arteries were thyroid superior arteries in 122 patients (79.22%), followed by external carotid arteries in 24 (15.58%), facial arteries in five (3.25%), and lingual arteries in three cases (1.95%). Venous anastomosis was performed in 109 patients (70.78%) as a termino-terminal suture between the thoracodorsal and external jugular veins. Forty-five (29.22%) patients underwent termino-lateral anastomosis between the thoracodorsal and internal jugular veins. 

Complications occurred in 35 patients (22.72%), 11 of whom required reoperation. Major postoperative complications occurred in 13 patients (8.44%) who developed perfusion impairment of the flap that caused necrosis of the skin paddle in nine (5.84%) and necrosis of the bone graft in four cases (2.60%). Among the patients who had minor complications (22.72%), 21 (13.63%) had wound dehiscence, seven (4.54%) had fistulas, three (1.95%) had bleeding, three (1.95%) had seroma, and one patient (0.65%) had lymphedema of the upper arm.

Furthermore, 45 patients (29.22%) with prolonged inability to feed orally required permanent percutaneous gastrostomy tube insertion.

Finally, 47 patients (30.51%) received fixed dentures in the edentulous mandible supported by four implants placed in the interforaminal region.

The overall five-year survival rate for HNSCC is 62.5% (*n* = 96).

That means that 62.5 of every 100 are still living five years after diagnosis. Conversely, 37.5 out of every 100 are dead within five years of an HNSCC diagnosis at our department.

In addition, the overall five-year survival rate is 69.56% for females and 60.49% for males.

A total of 18.18% of patients (*n* = 28) were presented with stage II, 14.29% (*n* = 22) with stage III, and 67.53% (*n* = 104) with stage IV HNSCC. 

There were 56 patients (36.36%) who had a tumor size from 2 cm to 4 cm in its greatest dimension (T2), 14.29% had a tumor size larger than 4 cm in its greatest dimension (T3), and 49.35% had a tumor growing into nearby structures such as bone (T4). Thirty-one patients (20.12%) were presented with no regional nodal disease (cN0). The presence of nodal disease rose as tumor size increased. No cases (M0) presented distant metastasis initially.

**Figure 1:**
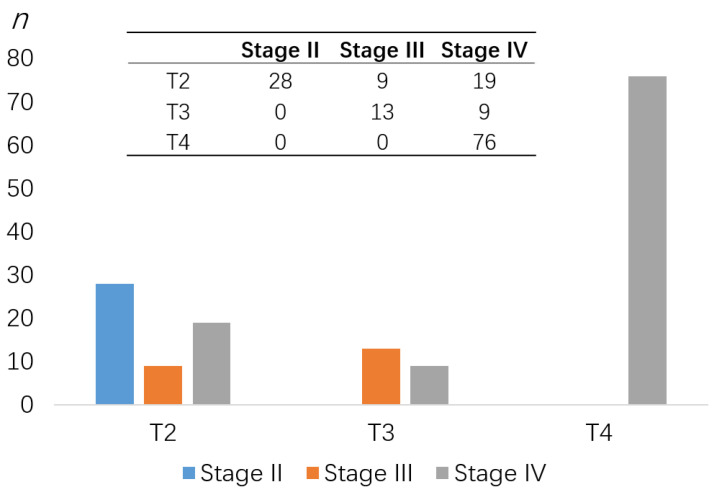
Patients’ cohort TNM classification according to the UICC [22].

QoL results: Frequency tables (Table 1 and Figure 1) of summary data (mean, standard deviation, median, and interquartile range) are provided for each of the nine multi-item scale scores and six single-item symptom scales of the EORTC Quality of Life Questionnaire Core 30 (QLQ-C30). A high score for cognitive functioning (mean 77.8) represented a high level of healthy cognitive functioning. The score for fatigue (mean 47.0) was highest among the symptom scales. Global health status and QoL had a mean of 59.4. A recent study reported a mean global health score of 64.4 [21].

This study defined QoL improvement as an increase of eight points in the mean score. Therefore, we assumed that the mean score was comparable with our results as it was within the range of the reference data.

**Figure 2:**
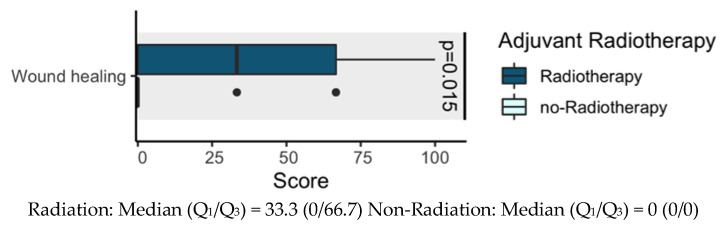
Influence of adjuvant therapies (radiotherapy) on quality of life (QoL).

We assessed interaction between radiotherapy and wound healing. Therefore, we completed a t-test for comparison between these variables. Results showed interaction between radiotherapy and wound healing (*p* = 0.015). Given the potential adverse effects that radiotherapy has on wound healing, we noted that there was treatment burden, possibly because of wound hypoperfusion, that delayed wound healing processes. However, a low mean scale score for wound healing is provided in Figure 2. No *p* values are reported for other outcomes. 

For more details and to increase familiarity with the scores for each scale, see Table 1 and Table 2.

## 3. Discussion

According to Bak et al., the goals of mandibular reconstruction comprise of the re-establishment of the form of the lower third of the face, and facilitation of oral intake and be intelligible while maintaining an unencumbered airway. Reconstruction of the outer and inner lining while maintaining its mobility is more critical to postoperative functional recovery than the reconstruction of the bony defect itself. Furthermore, restoring mandibular continuity while maintaining a proper occlusal relationship should provide a structure to produce and withstand the masticatory forces. This, in combination with dental rehabilitation, enables the re-establishment of a proper postoperative form and function of the lower third of the face, restoring an acceptable QoL [23].

The aim of this study was to demonstrate and share our clinical expertise in using the SFF for more than 35 years. In the following paragraphs, we discuss the various characteristics of the SFF as opposed to the DCIA flap and FFF. 

### 3.1. The Pedicle

With reference to the pedicle, our experience in the last 35 years confirms the distinct versatility of the SFF pediculated on the angular branch. This method significantly improves the length of the scapular pedicle, enhancing the arc of rotation of the bony flap and the mobility of the latissimus dorsi related to the scapula [5,9,12,24]. In a study involving 135 cadavers, Seitz et al. reported a mean length of 167 mm (range: 124–242 mm) when the subscapular artery was included [11]. Such a pedicle length enables contralateral anastomosis without the need for vein grafting (never used in this survey), thus representing a workhorse for the reconstruction of composite defects after salvage surgery, the treatment of osteoradionecrosis and after RND.

**Figure 3:**
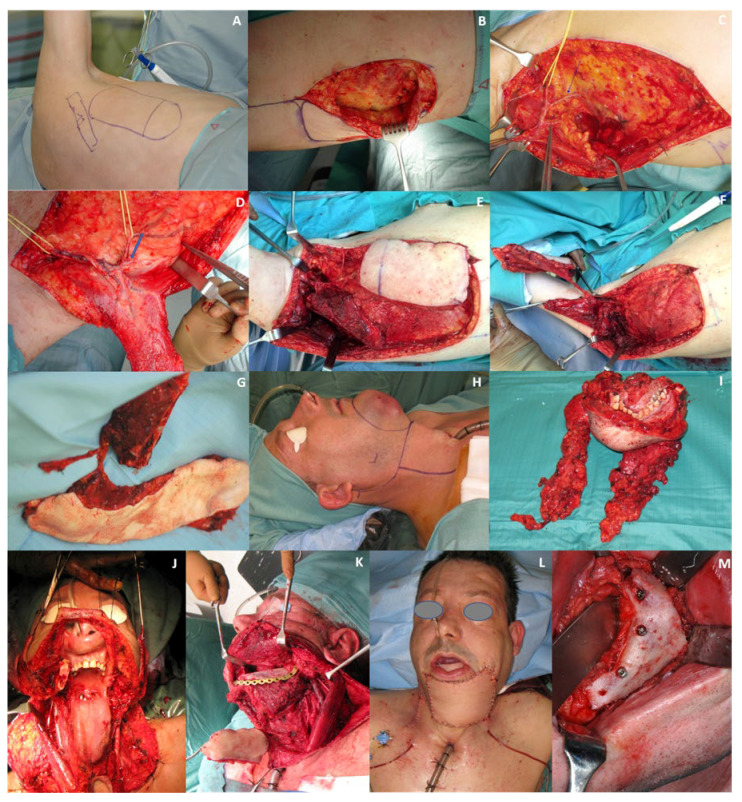
Raising of the subscapular system free flap (SFF): (**A**) Preoperative measuring for the skin paddle and bony transplant; (**B**) Raising of the skin paddle; (**C**) Intraoperative view showing the anatomy of the subscapular system during dissection of the SFF. The inferior angle of the scapula has been detached from the subscapular muscle and is displaced laterally with a towel clamp. The subscapular vessels, the circumflex artery (green arrow) and vein (white arrow), thoracodorsal vessels (vessel loop), the branches for the serratus muscle (blue arrow), and the angular branch (tweezers) were identified and preserved. In this case, the angular branch originated from the thoracodorsal artery. Clipping the circumflex artery and the branch for the serratus muscle was performed after complete dissection of the angular branch; (**D**) Depiction of the thoracodorsal vessels with the angular branch (blue arrow) after raising of the myocutaneous transplant; (**E**) Raising of the bony transplant of the SFF; (**F**) Depiction of the osseous (one-single hook) and myocutaneous transplant (fine double hook) after flap raising; (**G**) Raising of SFF completed; (**H**) Forty-eight-year-old male patient with oral cavity squamous cell carcinoma; (**I**) Specimen after tumor resection and neck dissection; (**J**) Intraoperative view of large defect after resection; (**K**) Reconstruction of the mandible; (**L**) Patient after complete defect reconstruction; (**M**) Intraoperative view of dental implants placed for oral rehabilitation 12 months postoperatively.

In our opinion, an additional advantage of flaps from the scapular region is the vessels’ gauge of the pedicle. According to Seitz et al., the gauge of the artery can vary between 3 mm (subscapular artery) and 1.3 mm (thoracodorsal artery) [11]. This wide range enlarges the choice of the recipient vessels suitable for end-to-end anastomosis from the thinnest branches of the external carotid artery to its main trunk. The two main advantages demonstrated by this pedicle are its length and gauge. Thus, the caliber of the pedicle can be adapted to that of the recipient vessels to avoid discrepancies. Furthermore, the surgeon can easily perform an end-to-end anastomosis. In the present study, the main trunk of the carotid artery was used as a recipient vessel in 24 patients (15.58%). In our opinion, one indication for this anastomosis was the need for a strong blood flow to revascularize a significant amount of tissue. Accordingly, among 24 patients who received termino-terminal anastomosis between the subscapular and external carotid arteries, in seven (4.54%), the length of the bony flap was ≥10 cm, while in 17 (11.04%), the skin paddle was >100 cm^2^. The biggest advantages here are shown in the consistent anatomy of the pedicle, pedicle length, and gauge (Figure 3). This could especially be an issue in patients undergoing preoperative or radiation procedures in the head and neck region, and extends to the vascular tissue quality, particularly for patients with a peripheral arterial occlusive disease or venous thrombosis in their medical record. These frequent comorbidities illustrate further limitations when considering FFFs or DCIA flaps for microsurgical reconstruction, and thereby would be accompanied by an inevitable increased risk of flap loss. Mandatory preoperative evaluation of vascular anatomy of the donor site in cases of using FFFs or DCIA flaps is also negligible, accompanied by factors such as time, cost, and any radiation-consuming diagnostic procedures, such as angiography [25,26]. Nevertheless, if considering FFFs or DCIA flaps for the mandible reconstruction, the pedicle length is still limited to 4–8 cm in both flaps, sometimes requiring interpositional vein grafts [27].

In fact, 20% of the patient cohort considered for an FFF procedure had to be excluded due to vascular comorbidities [28].

### 3.2. The Bony Graft

**Figure 4:**
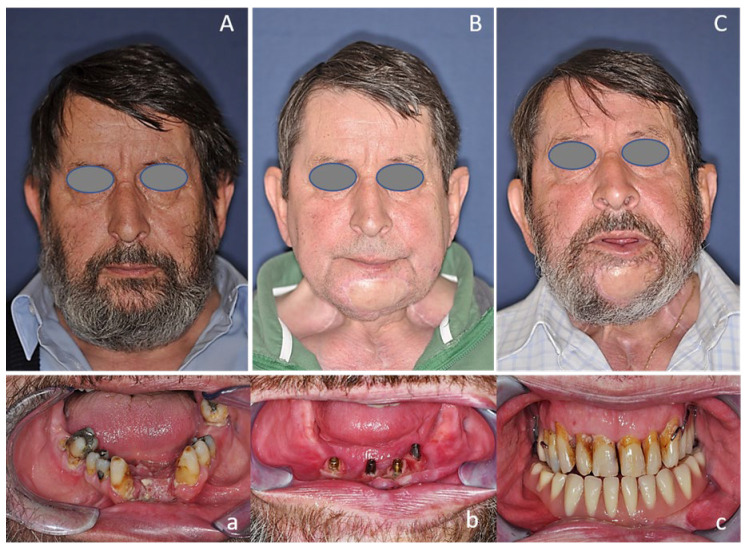
Fifty-seven-year-old male patient with oral cavity squamous cell carcinoma of the alveolar crest reconstructed with the subscapular system free flap. (**A**,**a**) Preoperative status; (**B**,**b**) Two years postoperatively with dental implants placed; (**C**,**c**) Five years postoperatively with implant-supported final prosthetics still placed.

Regarding the bony graft, we found that the length of the harvestable bone was 4–15 cm (mean: 7 cm). This allowed for the reconstruction of bone defects involving 1–3 mandibular segments in 145 (94.16%) patients. In seven patients (4.54%), it was even possible to reconstruct defects extending from one mandibular condyle to the contralateral mandibular ramus (four segments), without the need for a second bone-containing free flap. Moreover, four patients (2.60%) required a second flap from the iliac crest to bridge composite defects involving four, five, and six mandibular segments, respectively. The vertical height of the SFF can be improved by harvesting part of the infraspinatus fossa. This adds support to the mental anatomical region, contributing to the re-establishment of the form of the lower third of the face. We found this issue aesthetically relevant in 33 patients (21.42%) who underwent reconstruction of the entire mandibular symphysis (Figure 4). 

Except for the significantly greater length, due to the low height in both the FFF and SFF, an atrophied mandible can be reliably compensated using the double-barrel technique, not exclusively with the FFF, but also with the SFF [6,29].

When utilizing the FFF, it is mandatory to be aware of hematoma development from the osseous resection margins of the donor site, as well as consecutive compartment syndrome [30].

In contrast to the SFF, the FFF represents the longest bone available for tissue transfer, whereas the DCIA flap has the most adequate contour for reconstructing regions of the mandibular angle and symphysis. The DCIA, with its extensive bony tissue, enables various design options. Nevertheless, in contrast to the SFF, it is clearly limited in its range by enabling the possible reconstruction of the mandible up to half at most [31]. However, described frequencies of accompanied complications such as herniation (9.7%), long-lasting pain (8.4%), neuropathy (4.8%), and impotence (1.2%) when using the DCIA must be considered [32].

### 3.3. The Skin Graft

**Figure 5:**
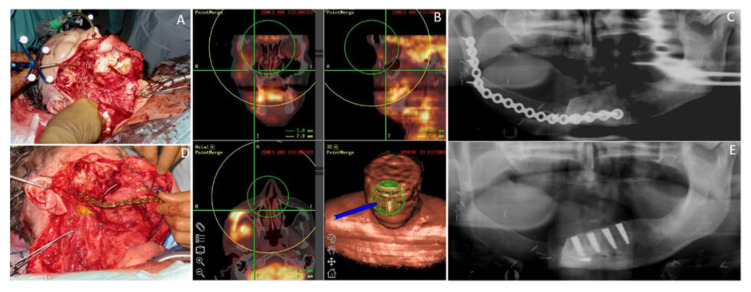
Fifty-seven-year-old male patient with a squamous cell carcinoma of the oral cavity (OCSCC) affecting the mandible reconstructed with the subscapular system free flap. (**A**) Intraoperative view of a large defect after navigated tumor resection; (**B**) Depiction of the 3D-navigation system based on PET/CT image-fusion, used perioperatively; (**C**) Postoperative dental panoramic radiograph after composite mandible reconstruction with the SFF. (**D**) Intraoperative view after insertion and fixation of the bony transplant of SFF reconstructing the mandible; (**E**) Postoperative dental panoramic radiograph after insertion of the dental implants.

Regarding the skin graft, the results of the present study confirm that a large amount of soft tissue is available [9]. The area of the skin paddle of the latissimus dorsi was 14–250 cm^2^ (mean 86.8 cm^2^) This large amount of skin and muscle allows not only closure of the intraoral defect, but also reconstruction of the bulk of the tongue, and good postoperative mobility of the remaining tongue. It is possible to harvest a muscle island bigger than the skin one, enabling closure of large intraoral defects by surrounding the reconstructive plate with revascularized muscular tissue. This issue can be crucial in osteonecrosis of the lower jaw, presenting factors that affect exposure of the reconstructive plate, such as skin fistulas or scars from previous neck procedures running along the mandibular border. Moreover, the independence of the components makes the SFF suitable for the reconstruction of mandibular defects associated with the oropharynx, tongue, cheek, or palate. In these cases, the skin overlying the muscle can be divided into two paddles, making the closure of trough-like defects feasible without the need for a second skin-containing free flap (Figure 5). After flap raising, no problems were observed with direct closure of the skin in all patients. However, after raising the FFF, direct closure of the skin is only achievable up to a flap width of 6–7 cm in the upper and middle third of the lower leg. In the distal third, skin grafts must almost always be used for covering the wound [33]. Utilizing osteomyocutaneous DCIA flaps is often accompanied by a skin paddle that is too voluminous for intraoral reconstructions [34]. Many authors recommend a second venous anastomosis in addition to the superficial venous system to ensure a higher certainty of survival of the very sensitive skin paddle, which otherwise would only be supplied by the deep venous system [35,36].

### 3.4. Dental Rehabilitation

**Figure 6:**
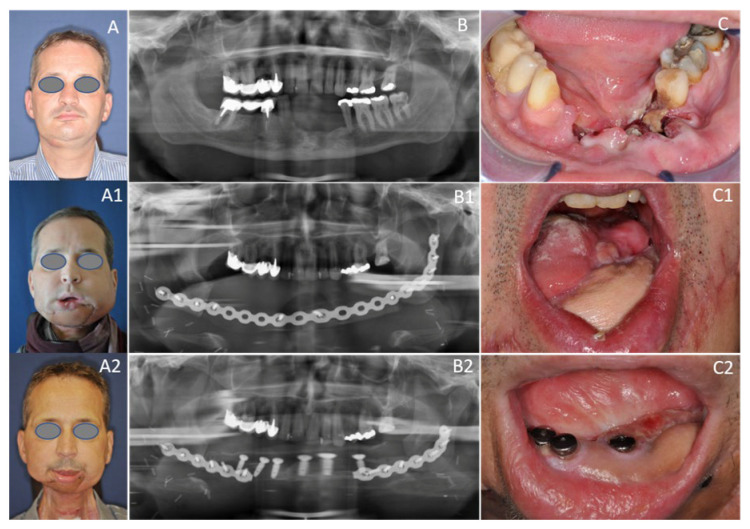
Fifty-four-year-old male patient with OCSCC reconstructed with the subscapular system free flap. (**A**) Preoperative status with perforating tumor on the right side; (**B**) Preoperative dental panoramic radiograph with large mandibular defect; (**C**) Preoperative clinical view with large OCSCC of the alveolar crest; (**A1**) Postoperative frontal view 10 days postoperatively; (**B1**) Postoperative dental panoramic radiograph displaying reconstructed mandible after insertion of the SFF. (**C1**) Postoperative clinical view enorally depicting the skin graft after composite mandible reconstruction with the SFF; (**A2**) Postoperative frontal view two years postoperatively; (**B2**) Postoperative dental panoramic radiograph displaying the mandible after insertion of implants; (**C2**) Postoperative clinical view enorally with placed implants.

Patients received fixed dentures in the edentulous mandible supported by four implants placed in the interforaminal region. Oral rehabilitation based on osseointegrated dental implants can often be difficult, as head and neck cancer patients show disability, facial deformity, and impaired speech after mandibular resection and reconstruction, and suffer from side effects of radiotherapy [37]. Dental implants are central to the sustainability of oral health and prosthetic rehabilitation, as the use of implant-retained prostheses facilitate chewing and speaking, and may provide aesthetic improvement [37]. Implant survival differs significantly across a variety of bone types, both resected and non-resected. According to the literature, implant survival was highest for implants placed into vascularized bone grafts, such as SFFs (100%), followed by FFFs (83%), while it was lowest in DCIA flaps (76%) [38]. This is in line with the findings of the present study that revealed remarkably high (100%) survival for implants placed into SFFs (*n* = 25). This substantial implant survival rate could be due to more cortical bone, as peri-implant bone resorption increases with lower cortical bone quality [38]. This confirmed that the lateral border of the scapula offers good bone quality and reliable osseointegration [39,40,41], contrary to preliminary reports [34]. Implant success, however, is usually reported to be lower than implant survival in the literature [37]. Furthermore, the comparison of implant success is difficult, as there are many different definitions in previous research and different implant systems used in oncologic patients [37]. In addition, the present study did not investigate radiographic peri-implant bone loss [37]. Interestingly, in the present sample, 22.7% of patients had dental rehabilitation (Figure 6) after discharge. There is evidence in the literature to support the observation that the use of dental implants in oncologic patients was relatively low, although dental rehabilitation was beneficial for patients [34,37,38].

Currently, there is limited information regarding the osseointegration, radiographic bone loss and success of dental implants in SFF. Previous studies, mainly focusing on radiographic bone loss, showed a relatively high rate of bone resorption in SFF, however the effect of the observed bone loss on dental implants remains uncertain [42,43]. This opens the question as to whether the SFF bone volume is adequate for dental implantation. One retrospective study found that bone height and width loss in SFF was significant one year after reconstructive surgery, but in this study only six patients received dental implants. Moreover, this study lacked coherence of the study population [42]. A more recent retrospective study, which included five patients with scapula free flaps, compared bone volume loss in several free flaps, but oral rehabilitation based on dental implants was not performed within two years of observation [43]. Interestingly, the same study reported that 3D volume analysis was more reliable than height-by-width measurements. In general, both studies regarded the scapula free flap as a good bone stock for dental implantation. Nevertheless, more studies will be required to further evaluate important aspects of the transferred scapula bone quantity and quality affecting dental rehabilitation in oncologic patients.

Based on the statements above, a crucial issue influencing the functional outcome of the reconstruction of composite defects of the oral cavity is the quantity and quality of soft tissue that can be transferred in combination with the bone.

### 3.5. Quality of Life

About two out of three patients with head and neck cancer receive adjuvant therapies such as radio- and/or chemotherapy [44], confirmed by observations in our study, where this was true for 29 out of 44 patients (68%). Studies have shown that irradiation has a negative effect on certain domains of QoL. Weight, salivary function, and physical function decreased significantly, whereas symptoms such as coughing, swallowing problems, and a dry mouth increased [45,46,47]. Our study showed similar results, but the differences were significant only on the wound healing disorder scale, as this symptom was more pronounced in adjuvant-irradiated patients. 

This statement underlines once again that the time at which patients are asked about their QoL is crucial. As already mentioned, these values fluctuate, especially in the first year; Pierre et al. agreed with Schliephake, Bozec, and Rogers et al. that the survey of QoL one year after treatment is a good long-term indicator [48,49,50].

Particularly advanced tumor stages (T3, T4) often require more extensive surgery and adjuvant therapy approaches, from which it can be deduced that an increasing tumor stage can have a negative effect on QoL [51].

Pierre et al. were able to show that the extent of the primary tumor had an influence on QoL, in a study in which there was a significant negative correlation in the scales of general health and quality of life for tumor stages T3 and T4, compared to less advanced stages (T1, T2). This also had an impact on certain symptoms, but no precise information on the scales was given [52].

Our study contains the following limitations: due to the small sample size as well as versatile confounders, which can influence QoL, further investigations are urgently necessary to be able to deliver conclusive statements; the determination of further factors influencing QoL, such as postoperative complications, as well as the definition of uniform time points of the survey, are interesting aspects that should be considered in further studies on similar topics.

Measurement of the pedicle length was not performed, although it could be of some value due to only single existing studies dealing with that topic. We did not investigate donor site morbidity. Nevertheless, we had the subjective impression that the mobility of the operated shoulder was similar to that of the contralateral arm. This impression appears to agree with other studies published in the past decade, but needs confirmation through standardized clinical trials and statistical evaluation [12,53]. Standardized investigations of postoperative swallowing and statistical comparisons with alternative reconstructive methods were also not objects of this study.

## 4. Materials and Methods 

### 4.1. Patients

Patients included in this retrospective single-center study were treated at the Department of Oral and Maxillofacial Surgery in Graz, Austria, between December 1983 and December 2019. All of the HNSCC patients had a diagnosis of oral cavity squamous cell carcinoma (OCSCC) that measured 2 cm or more. All patients underwent mandibular composite reconstruction with SFFs. Additional surgical procedures included radical, modified radical, or selective neck dissections. Exclusion criteria were an uncertain diagnosis of HNSCC, histological diagnosis of other malignancies, osteoradionecrosis, other reconstructive surgical procedures, and missing data on diagnosis or treatment.

This retrospective study was conducted in accordance with the moral, ethical, regulatory, and scientific principles governing clinical research as set out in the Declaration of Helsinki (2004), and approval was obtained from the appropriate institutional review boards. Ethical approval for the retrospective study was obtained by the local ethics committee (EK No. 30-431 ex 17/18). 

### 4.2. Quality of Life Assessment

#### 4.2.1. The EORTC QLQ-C30

A subgroup of patients filled out the validated cancer-specific EORTC QLQ-C30 (European Organization for the Research and Treatment of Cancer Quality of Life Questionnaire Core 30, version 3.0) and the latest module for patients regarding Head and Neck procedures, the EORTC QLQ-H&N43, at the time of scheduled follow-up visits between December 2018 and December 2019. 

All patients provided written informed consent to the application of all questionnaires after detailed explanation and discussion. Ethical approval for the amendment including quality of life assessment as part of the ongoing study was obtained by the local ethics committee (EK No. 31-119 ex 18/19).

The EORTC QLQ-C30 is a self-reported instrument, which encompasses 30 items. It measures five functional domains (which include physical, cognitive, role limitations, social function, emotional problems) and nine symptoms (which include bodily pain, fatigue, nausea/vomiting, constipation, diarrhea, insomnia, dyspnea, financial difficulty and appetite loss). In addition, it incorporates a global health perception and quality-of-life scale. Scores range from 0 to 100, with higher scores indicating better function and health status.

#### 4.2.2. The EORTC QLQ-H&N43 

The EORTC QLQ-H&N43 consists of 43 questions with 4 possible answers, each with the options “not at all”, “little”, “moderate” and “very”. This answer format is also called the 4-point Likert scale. During the evaluation, as with the QLQ-C30 questionnaire, groups of questions were formed in the form of scales, with scale values ranging from 0 to 100. As with the symptom scales of the QLQ-C30, a high value correlated with a high degree of symptomatology and with a lower quality of life. The 19 symptom scales of the QLQ-H&N43 are made up of 12 scales consisting of several questions (so-called multi item scales) and 7 single item scales.

Compared to the previous version, the updated H&N43 includes additional questions that specifically address side effects of newer cancer treatment strategies. These include skin problems, shoulder problems or neurological side effects [54].

The EORTC QLQ-C30 and QLQ-H&N43 questionnaires were assessed directly from patients at different time-points after surgery. Questionnaires were administered in the validated German translation or the patient’s native language.

### 4.3. Data Collection

Baseline measures, including personal data of all patients, demographic characteristics such as sex and age and clinical data such as histopathological diagnosis, were collected. In addition the extent of mandibulectomy, concomitant procedures such as neck dissection or additional flaps, the length of the bony graft, the dimensions of the skin paddle, the recipient vessels, and type of anastomosis (termino-lateral/latero-lateral), complications and radiotherapy were also registered. The difference in complications in our study was driven by two locations, including the donor or graft site, and two severity classifications including minor or major, according to the classification of Singh et al. [55]. Quality-of-life measures were obtained by means of written surveys at different time-points postoperatively.

### 4.4. Statistical Analysis

All the outcomes of the retrospective study are reported with descriptive statistics. Baseline characteristics are reported as means or frequencies and percentages, where appropriate.

All the analyses were performed with the use of SPSS Statistics (IBM SPSS Statistics for Windows, Version 25.0. Armonk, NY, USA).

#### Statistical Evaluation of the EORTC Questionnaires

According to the EORTC Scoring Manual the first step of the statistical evaluation is to collect an average of all given answers per scale [56]. The EORTC calls this value the Raw Score. 

Firstly, the achieved points of the answers from the questions within a scale are added and then divided by the total number of questions of this scale. For the individual questions, the raw score is also the number of points of the given answer.

(1) $$\mathrm{RS}=\left(I_{1}+I_{2}+I_{3}+\ldots +I_{n}\right)/n$$

Formula (1): Calculation of the Raw Score.

Now, a linear transformation is used to convert and standardize these average values to obtain a comparable scale with values from 0–100. This serves for a better illustration of the values. On this transformed scale, a high value (closer to 100) represents a higher or better function level for the function scales. On the Global Health and Quality of Life scale, high values also represent a high estimation of the quality of life. The opposite is true for the symptom scales, with high values representing a high or poor level of symptoms. The following formula is therefore used for the function scales:

(2) $$S=\left\{1-\frac{\left(\mathrm{RS}-1\right)}{\mathrm{range}}\right\}\times 100$$

S: Score; RS: Raw Score; Range: RS_max_ − RS_min_

Formula (2): Calculation of transformed linear scale value: function scales

Range is defined as the difference between the maximum and minimum possible values of the Raw Score. Most questions have a range of 3, since answers can be given on a scale from 1 to 4. The questions in the category “Global state of health and quality of life” result in values from 1 to 7, giving a range of 6.

For the symptom scales and the scale “Global state of health and quality of life” the result is

(3) $$S=\left\{\frac{\left(\mathrm{RS}-1\right)}{\mathrm{range}}\right\}\times 100$$

S: Score; RS: Raw Score; Range: RS_max_ − RS_min_

Formula (3): Calculation of transformed linear scale value: Symptom scales

The function scales must be reversed because the questions about the function in the QLQ-C30 are formulated in such a way that the higher the number of points in the raw score, the more severe the restriction in the function. For reasons of comparability, however, the transformed linear scale should indicate a better function level and higher quality of life at higher scores (closer to 100), which is the case after the polarity reversal. In the case of the symptom scales, however, lower scores (closer to 0) should indicate a better symptom level with a low level of symptoms. 

Interpretation of the scale values after linear transformation:

- High values in the function scales: high/better function level and low level of restriction
- High scores on the Global Health and QoL scale: high quality of life
- High values in the symptom scales: high/worse symptom level and high level of complaints

## 5. Conclusions

This present study provides evidence for the benefits of the SFF for composite mandibular reconstruction and rehabilitation of HNSCC as the treatment of choice. In particular, for patients who have large composite defects, the SFF is the gold standard. For this strategy to be successful, extensive experience to minimize flap failure is important. In accordance with the existing literature, we can confirm that intensified flap monitoring and early-stage revision enhances the flap outcome [57]. Our clinical experience supports the low morbidity or donor site complication rate using this technique [58].

In addition, our data show that applying this reliable functional reconstruction method in combination with the oral rehabilitation consecutively led to a better outcome in QoL.

Currently, there are no preliminary studies assessing QoL in these patients. Although our data on QoL are encouraging, more research is clearly needed to determine QoL improvement.

## Figures and Tables

**Table 1:** Results of the European Organization for Research and Treatment of Cancer Quality of Life Questionnaire Core 30 (EORTC QLQ-C30).

EORTC QLQ-C30	Median (Q_1_/Q_3_)	Mean ± SD
*Multi-item functional scales*		
Physical functioning	66.7 (40/75)	63.2 ± 22.7
Role functioning	50 (16.7/66.7)	50.0 ± 32.7
Cognitive functioning	83.3 (66.7/100)	77.8 ± 25.7
Emotional functioning	75 (41.7/83.3)	66.7 ± 26.4
Social functioning	58.3 (16.7/70.8)	47.6 ± 33.2
*Multi-item symptom scales*		
Fatigue	44.4 (22.2/66.7)	47 ± 25.5
Bodily pain	33.3 (16.7/33.3)	34.4 ± 29.2
Nausea/Vomiting	0 (0/16.7)	6.3 ± 10.3
*Single item symptom scales*		
Dyspnea	33.3 (0/33.3)	27.1 ± 30.4
Appetite loss	16.7 (0/33.3)	23.8 ± 30.5
Sleep disturbance	33.3 (0/66.7)	41.7 ± 39.4
Constipation	16.7 (0/58.3)	29.2 ± 36.3
Diarrhea	0 (0/25)	8.3 ± 14.9
Financial difficulty	33.3 (0/66.7)	35.6 ± 34.4
Global health status and quality of life	50 (41.7/75)	59.4 ± 20.9

**Table 2:** Results for EORTC QLQ-H&N43.

EORTC QLQ-H&N43	Median (Q_1_/Q_3_)	Mean ± SD
*Multi-item scales*		
Anxiety	33.3 (33.3/75)	49 ± 30.1
Skin problems	11.1 (0/22.2)	13.2 ± 11.6
Body image	27.8 (0/75)	36.8 ± 35.3
Dry mouth/sticky saliva	50 (50/66.7)	52.2 ± 26.6
Social eating	62.5 (14.6/83.3)	52 ± 36
Swallowing	50 (33.3/75)	53.7 ± 28.5
Pain in the mouth	16.7 (8.3/33.3)	25.6 ± 24.1
Shoulder problems	66.7 (16.7/83.3)	57.8 ± 35.6
Sexuality	50 (0/66.7)	42.4 ± 40.4
Sense problems	0 (0/33.3)	13.5 ± 20.4
Speech problems	51.7 (8.3/71.7)	46.5 ± 33.4
Teeth	72.2 (33.3/100)	63.1 ± 32.1
*Single-item scales*		
Weight loss	33.3 (0/33.3)	33.3 ± 32.2
Swelling in the neck	16.7 (0/58.3)	29.2 ± 36.3
Coughing	16.7 (0/33.3)	16.7 ± 17.2
Opening mouth	66.7 (33.3/83.3)	62.2 ± 30.5
Neurologic problems	16.7 (0/33.3)	25 ± 33.3
Social contact	0 (0/66.7)	33.3 ± 42.2
Wound healing	0 (0/66.7)	25 ± 31

## References

[1] Daniel R.K., Taylor G.I. (1973). Distant transfer of an island flap by microvascular anastomoses: A clinical technique. Plast. Reconstr. Surg..

[2] O’Brien B.M., Macleod A.M., Hayhurst J.W., Morrison W.A. (1973). Successful transfer of a large island flap from the groin to the foot by micro-vascular anastomoses. Plast. Reconstr. Surg..

[3] Baudet J., Guimberteau J.-C., Nascimento E. (1976). Successful clinical transfer of two free thoraco-dorsal axillary flaps. Plast. Reconstr. Surg..

[4] Swartz W.M., Banis J.C., Newton E.D., Ramasastry S.S., Jones N.F., Acland R. (1986). The osteocutaneous scapular flap for mandibular and maxillary reconstruction. Plast. Reconstr. Surg..

[5] Urken M.L., Bridger A.G., Zur K.B., Genden E.M. (2001). The Scapular Osteofasciocutaneous Flap: A 12-Year Experience. Arch. Otolaryngol. Head Neck Surg..

[6] Jones N.F., Swartz W.M., Mears D.C., Jupiter J.B., Grossman A. (1988). The “double barrel” free vascularized fibular bone graft. Plast. Reconstr. Surg..

[7] Granick M.S., Ramasastry S.S., Newton E.D., Solomon M.P., Hanna D.C., Kaltman S. (1990). Reconstruction of complex maxillectomy defects with the scapular–free flap. Head Neck.

[8] Van Thienen C.E., Deraemaecker R. (1988). The serratus anterior scapular flap—A new osteomuscular unit. Eur. J. Plast. Surg..

[9] Coleman J.J., Sultan M.R. (1991). The bipedicled osteocutaneous scapula flap: A new subscapular system free flap. Plast. Reconstr. Surg..

[10] Kärcher H. (1991). Transplantation of the scapular bone vascularized by the thoracodorsal vessels. A new method of scapula transplantation. Dtsch. Z. Mund Kiefer Gesichts Chir..

[11] Seitz A., Papp S., Papp C., Maurer H. (1999). The anatomy of the angular branch of the thoracodorsal artery. Cells Tissues Organs..

[12] Clark J.R., Vesely M., Gilbert R. (2008). Scapular angle osteomyogenous flap in postmaxillectomy reconstruction: Defect, reconstruction, shoulder function, and harvest technique. Head Neck.

[13] Brown J., Bekiroglu F., Shaw R. (2010). Indications for the scapular flap in reconstructions of the head and neck. Br. J. Oral Maxillofac. Surg..

[14] The World Health Organization (1995). Quality of Life Assessment (WHOQOL): Position Paper from the World Health Organization. Soc. Sci. Med..

[15] Dietz A., Meyer A., Singer S. (2009). Measuring quality of life in head and neck cancer: Current status and future needs. HNO.

[16] Egger J.W. (2005). Das biopsychosoziale Krankheitsmodell- Grundzüge eines wissenschaftlich begründeten ganzheitlichen Verständnisses von Krankheit. Psychol. Med..

[17] Karimi M., Brazier J. (2016). Health, Health-Related Quality of Life, and Quality of Life: What is the Difference?. Pharmacoeconomics.

[18] Daig I., Lehmann A. (2007). Verfahren zur Messung der Lebensqualität. Zeitschrift fur Medizinische Psychologie.

[19] Rogers S.N., Lowe D., Brown J.S., Vaughan E.D. (1998). A comparison between the University of Washington Head and Neck Disease-Specific Measure and the Medical Short Form 36, EORTC QOQ-C33 and EORTC Head and Neck 35. Oral Oncol..

[20] Urken M.L., Weinberg H., Vickery C., Buchbinder D., Lawson W., Biller H.F. (1991). Oromandibular Reconstruction Using Microvascular Composite Free Flaps: Report of 71 Cases and a New Classification Scheme for Bony, Soft-Tissue, and Neurologic Defects. Arch. Otolaryngol. Neck Surg..

[21] Scott N.W., Fayers P.M., Aaronson N.K., Bottomley A., De Graeff A., Groenvold M., Gundy C., Koller M., Petersen M.A., Sprangers M.A. (2008). EORTC QLQ-C30 Reference Values Manual.

[22] Brierley J.D., Gospodarowicz M.K., Wittekind C. (2017). UICC TNM Classification of Malignant Tumours.

[23] Bak M., Jacobson A.S., Buchbinder D., Urken M.L. (2010). Contemporary reconstruction of the mandible. Oral Oncol..

[24] Wagner A.J., Bayles S.W. (2008). The angular branch: Maximizing the scapular pedicle in head and neck reconstruction. Arch. Otolaryngol. Head Neck Surg..

[25] Pinto N.R., Ubilla M., Zamora Y., Del Rio V., Dohan Ehrenfest D.M., Quirynen M. (2018). Leucocyte- and platelet-rich fibrin (L-PRF) as a regenerative medicine strategy for the treatment of refractory leg ulcers: A prospective cohort study. Platelets.

[26] Taylor G.I., Miller G.D., Ham F.J. (1975). The free vascularized bone graft. A clinical extension of microvascular techniques. Plast. Reconstr. Surg..

[27] Van Twisk R., Pavlov P.W., Sonneveld J. (1998). Reconstruction of bone and soft tissue defects with free fibula transfer. Ann. Plast. Surg..

[28] Wolff K.D., Ervens J., Herzog K., Hoffmeister B. (1996). Experience with the osteocutaneous fibula flap: An analysis of 24 consecutive reconstructions of composite mandibular defects. J. Cranio-Maxillo-Facial Surg..

[29] Nkenke E., Vairaktaris E., Stelzle F., Neukam F.W., Stockmann P., Linke R. (2009). Osteocutaneous free flap including medial and lateral scapular crests: Technical aspects, viability, and donor site morbidity. J. Reconstr. Microsurg..

[30] Coghlan B.A. (1993). Townsend PLG The morbidity of the free vascularised fibula flap. Br. J. Plast. Surg..

[31] Bitter K., Danai T. (1983). The iliac bone or osteocutaneous transplant pedicled to the deep circumflex iliac artery. I. Anatomical and technical considerations. J. Maxillofac. Surg..

[32] Forrest C., Boyd B., Manktelow R., Zuker R., Bowen V. (1992). The free vascularised iliac crest tissue transfer: Donor site complications associated with eighty-two cases. Br. J. Plast. Surg..

[33] Wolff K.-D., Hölzle F. (2017). Raising of Microvascular Flaps.

[34] Stringer S.P., Mark L.U., Mack L.C., Michael J.S., Hugh F.B. (1994). Atlas of Regional and Free Flaps for Head and Neck Reconstruction.

[35] Harrison D. (1989). Reconstructive microsurgery. Br. J. Surg..

[36] Sanders R., Mayou B.J. (1979). A new vascularized bone graft transferred by microvascular anastomosis as a free flap. Br. J. Surg..

[37] Ettl T., Junold N., Zeman F., Hautmann M., Hahnel S., Kolbeck C., Müller S., Klingelhöffer C., Reichert T.E., Meier J.K. (2020). Implant survival or implant success? Evaluation of implant-based prosthetic rehabilitation in head and neck cancer patients—A prospective observational study. Clin. Oral Investig..

[38] Laverty D.P., Addison O., Wubie B.A., Heo G., Parmar S., Martin T., Praveen P., Pearson D., Newsum D., Murphy M. (2019). Outcomes of implant-based oral rehabilitation in head and neck oncology patients—A retrospective evaluation of a large, single regional service cohort. Int. J. Implant. Dent..

[39] Moscoso J.F., Keller J., Genden E., Weinberg H., Biller H.F., Buchbinder D., Urken M.L. (1994). Vascularized Bone Flaps in Oromandibular Reconstruction: A Comparative Anatomic Study of Bone Stock From Various Donor Sites to Assess Suitability for Enosseous Dental Implants. Arch. Otolaryngol. Neck Surg..

[40] Akkocaoglu M., Cehreli M.C., Tekdemir I., Comert A., Güzel E., Daǧdeviren A., Akca K. (2007). Primary Stability of Simultaneously Placed Dental Implants in Extraoral Donor Graft Sites: A Human Cadaver Study. J. Oral Maxillofac. Surg..

[41] Schultes G., Gaggl A., Kärcher H. (2002). Stability of dental implants in microvascular osseous transplants. Plast. Reconstr. Surg..

[42] Lanzer M., Gander T., Grätz K., Rostetter C., Zweifel D., Bredell M. (2015). Scapular Free Vascularised Bone Flaps for Mandibular Reconstruction: Are Dental Implants Possible?. J. Oral Maxillofac. Res..

[43] Wilkman T., Apajalahti S., Wilkman E., Törnwall J., Lassus P. (2017). A Comparison of Bone Resorption Over Time: An Analysis of the Free Scapular, Iliac Crest, and Fibular Microvascular Flaps in Mandibular Reconstruction. J. Oral Maxillofac. Surg..

[44] Li W., Xu Z., Liu F., Huang S., Dai W., Sun C. (2013). Vascularized free forearm flap versus free anterolateral thigh perforator flaps for reconstruction in patients with head and neck cancer: Assessment of quality of life. Head Neck.

[45] Connor N.P., Cohen S.B., Kammer R.E., Sullivan P.A., Brewer K.A., Hong T.S., Chappell R.J., Harari P.M. (2006). Impact of conventional radiotherapy on health-related quality of life and critical functions of the head and neck. Int. J. Radiat. Oncol. Biol. Phys..

[46] Petruson K.M., Silander E.M., Hammerlid E.B. (2005). Quality of life as predictor of weight loss in patients with head and neck cancer. Head Neck.

[47] Epstein J.B., Robertson M., Emerton S., Phillips N., Stevenson-Moore P. (2001). Quality of life and oral function in patients treated with radiation therapy for head and neck cancer. Head Neck.

[48] Schliephake H., Jamil M.U. (2002). Prospective evaluation of quality of life after oncologic surgery for oral cancer. Int. J. Oral Maxillofac. Surg..

[49] Bozec A., Poissonnet G., Chamorey E., Casanova C., Vallicioni J., Demard F., Mahdyoun P., Peyrade F., Follana P., Bensadoun R.J. (2008). Free-flap head and neck reconstruction and quality of life: A 2-year prospective study. Laryngoscope.

[50] Rogers S.N., Lowe D., Brown J.S., Vaughan E.D. (1999). The University of Washington head and neck cancer measure as a predictor of outcome following primary surgery for oral cancer. Head Neck.

[51] de Melo N.B., Bernardino Í.d.M., de Melo D.P., Gomes D.Q.C., Bento P.M. (2018). Head and neck cancer, quality of life, and determinant factors: A novel approach using decision tree analysis. Oral Surg. Oral Med. Oral Pathol. Oral Radiol..

[52] Pierre C.S., Dassonville O., Chamorey E., Poissonnet G., Riss J.C., Ettaiche M., Peyrade F., Benezery K., Chand M.E., Leyssalle A. (2014). Long-term functional outcomes and quality of life after oncologic surgery and microvascular reconstruction in patients with oral or oropharyngeal cancer. Acta Otolaryngol..

[53] Coleman S.C., Burkey B.B., Day T.A., Resser J.R., Netterville J.L., Dauer E., Sutinis E. (2000). Increasing use of the scapula osteocutaneous free flap. Laryngoscope.

[54] Singer S., Amdal C.D., Hammerlid E., Tomaszewska I.M., Castro Silva J., Mehanna H., Santos M., Inhestern J., Brannan C., Yarom N. (2019). International validation of the revised European Organisation for Research and Treatment of Cancer Head and Neck Cancer Module, the EORTC QLQ-HN43: Phase IV. Head Neck.

[55] Singh B. (1999). Factors associated with complications in microvascular reconstruction of head and neck defects. Plast. Reconstr. Surg..

[56] Fayers P.M., Aaronson N.K., Bjordal K., Groenvold M., Curran D., Bottomley A., on behalf of the EORTC Quality of Life Group (2001). The EORTC QLQ-C30 Scoring Manual.

[57] Al-Dam A., Zrnc T.A., Hanken H., Riecke B., Eichhorn W., Nourwali I., Smeets R., Blessmann M., Heiland M., Gröbe A. (2014). Outcome of microvascular free flaps in a high-volume training centre. J. Cranio-Maxillofac. Surg..

[58] Kearns M., Ermogenous P., Myers S., Ghanem A.M. (2018). Osteocutaneous flaps for head and neck reconstruction: A focused evaluation of donor site morbidity and patient reported outcome measures in different reconstruction options. Arch. Plast. Surg..

